# Relative age and specific learning disorder diagnoses: A Finnish population‐based cohort study

**DOI:** 10.1111/jcv2.12001

**Published:** 2021-03-17

**Authors:** Bianca Arrhenius, David Gyllenberg, Miika Vuori, Elina Tiiri, Lotta Lempinen, Andre Sourander

**Affiliations:** ^1^ Department of Child Psychiatry University of Turku Turku Finland; ^2^ INVEST Research Flagship University of Turku Turku Finland; ^3^ Child and Youth Health Services City of Helsinki Helsinki Finland; ^4^ Children, Adolescents and Families Unit Finnish Institute for Health and Welfare Helsinki Finland; ^5^ Department of Adolescent Psychiatry Helsinki University Central Hospital Helsinki Finland; ^6^ Department of Teacher Education Turku Institute for Advanced Studies University of Turku Turku Finland; ^7^ Department of Child Psychiatry Turku University Hospital Turku Finland

**Keywords:** arithmetic disorder, birth month, learning disorders, neurodevelopmental, reading disorder, relative age

## Abstract

**Background:**

Being among the youngest in class has previously been associated with attention‐deficit/hyperactivity disorder (ADHD) and academic disadvantage, but the relative age effect on learning disorders is less well understood. This study examined whether relatively young children are more likely to be diagnosed with specific learning disorders than their older peers.

**Methods:**

The setting included all 388,650 children born singleton in Finland from 1996 to 2002. Cases diagnosed with specific learning disorders in specialized health care by the age of 10 were identified from national registers. Cumulative incidences of specific learning disorders and the corresponding incidence rate ratios (IRRs) and 95% confidence intervals (CIs) were calculated for each birth month compared to January.

**Results:**

During follow‐up, 3162 (0.8% of 388,650) children were diagnosed with a specific learning disorder. Children born in December displayed higher cumulative incidences for specific learning disorders than children born in January (IRR: 1.77, 95% CI: 1.50–2.11). The findings were similar for girls (IRR: 2.01, 1.44–2.83) and boys (IRR: 1.70, 1.39–2.08). ADHD did not explain the association, as the IRR for the youngest children with specific learning disorders and ADHD was 1.59 (1.13–2.26) compared to those without ADHD (IRR: 1.84, 1.51–2.24).

**Conclusions:**

Relatively younger children in Finnish schools were more likely to be diagnosed with a specific learning disorder by the age of 10. Increased awareness of how relative age differences affect the likelihood for children to be diagnosed with specific learning disorders is needed among parents, clinicians, and teachers.

## Graphical abstract

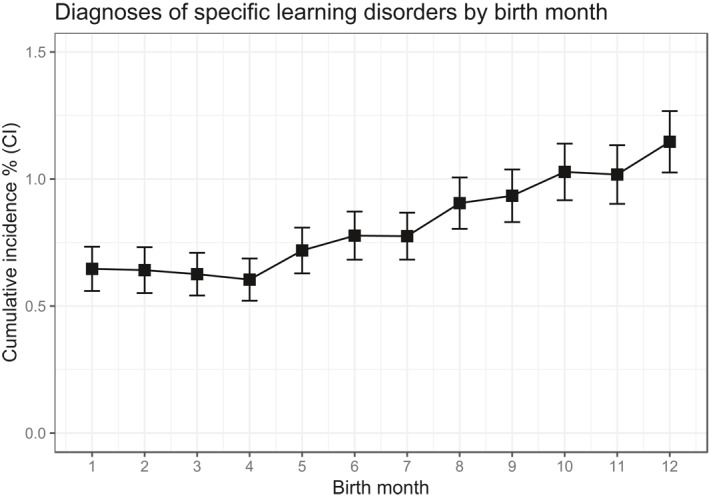

This population‐based study examined a source cohort of all 388,650 children born in Finland between 1996 and 2002, of whom 3,162 children were diagnosed with a specific learning disorder by the age of 10. The association between birth month and diagnoses of specific learning disorders was notable; the youngest children in class were almost twice as likely to be diagnosed with a specific learning disorder than their older peers, and comorbid ADHD did not explain the finding.

## 1 INTRODUCTION

The way that academic admissions are structured in many countries means that some children can be up to a year younger when they start school than other children in the same grade, depending on when their birthday is. Some studies have reported that the youngest children in classrooms can be more likely to display various adversities than their older peers. This issue, also known as the relative age effect, was first recognized as far back as the 1990s (Bell & Daniels, 1990). Later studies confirmed the association between young relative age and being diagnosed with attention‐deficit/hyperactivity disorder (ADHD)(Caye et al., 2019; Holland & Sayal, 2018; Whitely et al., 2019), other psychiatric problems (Goodman, 2003) and intellectual disabilities (Root et al., 2019). Younger age has also been associated with being bullied (Tiiri et al., 2020).

**Key points**

- Being among the youngest in class has previously been associated with psychiatric disorders, low academic achievement, and being bullied.
- No previous studies on the association between clinically diagnosed specific learning disorders and relative age in a nationwide sample.
- We found that younger relative age among children in Finnish schools was associated with specific learning disorder diagnoses by the age of 10 in specialized healthcare. Furthermore, the association was not explained by comorbid attention‐deficit/hyperactivity disorder.
- From a clinical perspective, the findings indicate that professionals should reflect on the relative age of a child when considering the possibility of a learning disorder.
- From an educational perspective, greater flexibility in school admissions for less mature children might be warranted.

However, it is unclear if relatively younger children are more likely to be diagnosed with specific learning disorders. This term refers to children who have difficulties with reading, spelling, or arithmetic skills, despite the fact that their overall intellectual functioning is within the normal range. Learning disorders are diagnosed with the help of age‐adjusted standardized psychological tests. In theory, as the diagnostic procedures acknowledge the child's exact age, the risk for unwarranted diagnoses due to their relatively young age should be minimized. A relative age effect for learning disorder diagnoses would have important implications for current practices, from correct referral processes in schools to clinical diagnostic evaluations. Moreover, knowledge on the topic is crucial for educational policy making, such as whether immature children should start school later than their relatively older peers.

Large studies on learning and education have reported that the relatively young children in school grades were more likely to need special education (Dhuey & Lipscomb, 2010; Gledhill et al., 2002; Kivinen, 2018) and display lower academic performance (Zoëga et al., 2012). In contrast, two Dutch studies (Jeronimus et al., 2015; Wienen et al., 2018) found no association between relative age and academic performance. However, those surveys both excluded children who were receiving special education and one only included adolescents (Jeronimus et al., 2015). Two studies have examined relative age and learning disorders, and both found a positive association (Dhuey & Lipscomb, 2010; Martin et al., 2004). However, the learning disorder diagnoses were survey based, either parent‐ or school‐reported, which may have limited their validity. Two population‐based studies on relative age and ADHD examined the role of comorbid learning disorders. A Finnish register study (Sayal et al., 2017) found that comorbid learning and coordination disorders did not influence the relative age effect on being diagnosed with ADHD. An Italian study (The Lombardy ADHD Group, 2018) suggested similar results, but also found an increased number of neurodevelopmental and psychiatric diagnoses in children born later in the school year, regardless of whether they had comorbid ADHD. To conclude, studies on the association between relative age and specific learning disorders are scarce and somewhat inconclusive.

That is why we conducted a nationwide register‐based study of children born in Finland from 1996 to 2002. The main aim was to study the association between specific learning disorders and relative age within the school year. Our second aim was to assess how comorbid ADHD and other learning and coordination disorders influenced the relative age effect of being diagnosed with a specific learning disorder. Based on previous studies, we hypothesized that relatively young children would be over‐represented in those diagnosed with specific learning disorders, but that the effects might be partly explained by ADHD.

## 2 METHODS

### 2.1 Design and participants

The study included all 388,650 singleton live births in Finland between 1996 and 2002. The cohort was followed up until the age of 10, and we identified the children who were diagnosed with specific learning disorders by specialist health care services during this period. These diagnoses were based on the International Classification of Diseases, 10th Revision (ICD‐10) codes F81.x.

In Finland, all children undergo free, routine, primary care health check‐ups by a trained nurse once a year. They are also seen by a physician eight times during childhood: five times before entering school and then at the ages of 7, 11, and 14. If learning disorders are suspected, children are usually examined first by the school psychologist and then, if needed, referred to publicly funded specialist clinics for diagnosis. The diagnostic procedure is multi‐professional and based on standardized psychological tests (Arrhenius et al., 2018).

Finnish children start primary school in August of the calendar year that they turn seven. The oldest children, born in January, are 7 years and 7 months and the youngest, born in December, are 6 years and 7 months. Only 1–2% of pupils are held back from starting primary school during that calendar year and the trend has been declining. Data from Statistics Finland show that 1161 of all the 56,770 Finnish children Starting school were held back in 2009 (2.0%), compared to the more recent numbers from 2018, which had decreased to 665/61,296 (1.1%; Official Statistics Finland, 2019).

### 2.2 Registers

Our study used data from Finnish nationwide registers, which are maintained by the Finnish Institute for Health and Welfare. The Medical Birth Register (Haukka, 2009), which contains information on all births in Finland, supplied information on the number of live‐born singleton children, their sex and birth month. Diagnostic data were provided by the Care Register for Health Care (Sund, 2012), which contains data on inpatient care provided by all hospitals since 1969 and outpatient care provided by all public hospitals since 1998. The information we collected included the admission date, the main diagnosis, and any possible secondary diagnosis. Since 1996, all diagnoses are recorded according to the ICD‐10, which is the diagnostic system used in Finland. Primary care diagnoses and entries by school psychologists are not included in the register. The Care Register for Health Care is widely used in epidemiological research and previous studies have shown it has good validity for neuropsychological diagnoses, such as autism (Lampi et al., 2010), ADHD (Joelsson et al., 2016), and tic disorders (Leivonen et al., 2014). Diagnostic data were available up to December 31, 2012. The youngest study participants were born in 2002, which limited the maximum age at diagnosis to 10 years.

The unique personal identification code, which is assigned to all Finnish citizens, linked the data from the two registers. We then anonymized and handled the data according to Finnish data protection laws. No registered cases were contacted and, therefore, no informed consent was required. The data protection authorities gave us permission to use the register data and The Ethics Committee of the Hospital District of Southwest Finland provided ethical approval for the study (Registration number: THL/1803/5.05.00/2013).

### 2.3 Comorbidities

To examine the role of comorbid disorders, we stratified the specific learning disorder cases according to the presence of ADHD, and other learning or coordination disorders, into mutually exclusive groups. The comorbid ADHD group consisted of cases that were diagnosed using ICD‐10 codes F90.x and we compared them to cases without comorbid ADHD diagnoses. Similarly, the comorbid learning or coordination disorder group consisted of cases that were also diagnosed with speech, coordination, or mixed learning disorders (ICD‐codes F80.x and/or F82 and/or F83) and those were compared to cases without other learning or coordination disorders.

### 2.4 Statistical analyses

We compared the incidences of children diagnosed with specific learning disorders for each birth month, with the oldest children, born in January of each year, as baseline. First, we calculated the cumulative incidence of specific learning disorders for each birth month for the total sample, by pooling the birth years 1996–2002, and according to sex. Second, we calculated the cumulative incidence by using pooled age groups that each contained four months: January–April, May–August, and September–December. The numerator was the number of children with specific learning disorders and the denominator was the total number of children born during the corresponding period. We then estimated each incidence rate ratio (IRR) and 95% confidence interval (CI) using generalized linear regression, assuming a Poisson error distribution. IRRs in which the denominator of the incidence rate is defined as the average population during the specified time interval, instead of summed person‐years of observation, are commonly used in large epidemiological samples (Centers for Disease Control and Prevention, 2006). The total number of children born during the inclusion years 1996–2002 reflect the average population size satisfyingly well, as the yearly death rate among children under 10 years in Finland is very low, approximately 0.2% (Official Statistics of Finland, 2018).

The younger age groups were compared to the oldest group of children born in just January or in January–April in the pooled month analysis. To control for the possible effect of the birth year, we added it as a categorical predictor variable to the regression model. However, birth year was not significant and was not included in the final model. Next, we assessed the possible effect of comorbid disorders by estimating the cumulative incidence for children with and without comorbid ADHD and other learning disorders. We estimated IRRs for comorbid ADHD versus no comorbid ADHD and for those with and without comorbid learning or coordination disorders. We calculated IRRs separately for each birth month and for the pooled 4‐month periods. A level of *p* < 0.05 was statistically significant. We used R statistical software version 3.5.2 (R Foundation for Statistical Computing) to perform the analyses.

## 3 RESULTS

Of the 388,650 children born alive in Finland in 1996–2002, 3860 (1.0%) children were diagnosed with a specific learning disorder by the age of 10. We excluded 433 children diagnosed with intellectual disability and/or autism spectrum disorder, because these diagnoses conflict with the definition of specific learning disorder. To further enhance the validity of the diagnoses, we excluded 265 cases diagnosed with specific learning disorders before they started school, if their condition was not verified after that age. The final number of cases was 3162 (0.8%) with specific learning disorders.

**FIGURE 1:**
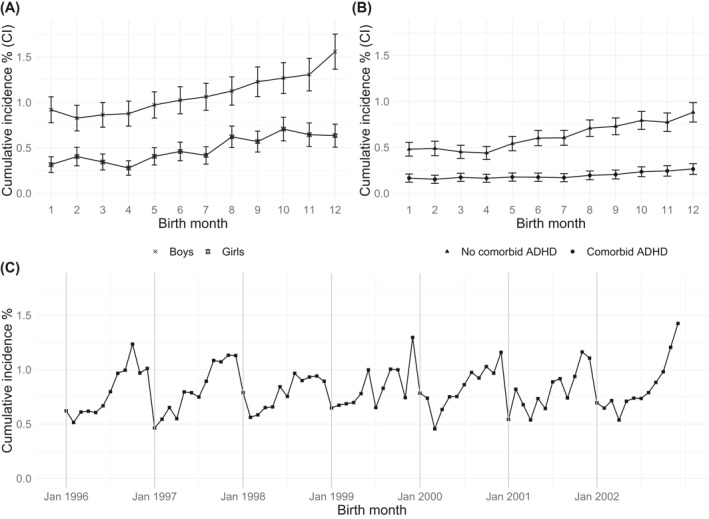
Cumulative incidences of specific learning disorders by: (A) birth month and sex, (B) birth month and with or without comorbid ADHD, and (C) by birth month and year. ADHD, attention‐deficit/hyperactivity disorder; CI, confidence interval; Jan, January

The median age at first diagnosis was 8.4 years (interquartile range: 7.3–9.2). Table 1 shows what month the 3162 cases with specific learning disorder were in born during the admission year and the number for each month ranged from 193 to 342, with the largest number born in December. Of these, 817 (26%) were born in January–April, 1073 (34%) in May–August, and 1272 (40%) in September–December. Overall, the IRRs for specific learning disorder diagnoses were higher for younger children born later in the school year than for older children born in January (Table 1). The youngest children, who were born in December, displayed the highest IRRs, with similar results for boys and girls (Table 1, Figure 1A). The IRR for the pooled 4‐month periods for both sexes was 1.64 (95% CI: 1.50–1.79, *p* < 0.001) for those born in September–December compared to those born in January–April (Table 1). In addition, children born mid‐year presented higher likelihoods for specific learning disorders diagnoses than the older children born at the start of the year, when both the month‐specific and pooled birth month analyses were examined (Table 1). The trend of cumulative incidences, which peaked towards the end of the year, when the children were younger, was similar regardless of the children's birth year (Figure 1C). The results were also similar when analyzing the whole sample prior to the exclusion of children with intellectual disability, autism spectrum disorder, or children diagnosed before the age of six; the IRR for December‐born children was 1.76 (1.51–2.06) compared to January‐born children in the total sample.

**TABLE 1:** Incidence rate ratios of specific learning disorder by the age of 10, by each birth month and the pooled birth years 1996–2002

	Both sexes			Girls		Boys	
Birth month(s)	Cases (3162)	No. of born	IRR (95% CI)	Cases (942)	IRR (95% CI)	Cases (2200)	IRR (95% CI)
January	210	32,480	Reference	52	Reference	158	Reference
February	193	30,088	0.99 (0.82–1.21)	61	1.28 (0.88–1.85)	132	0.89 (0.71–1.13)
March	212	33,888	0.97 (0.80–1.17)	59	1.08 (0.74–1.57)	153	0.93 (0.75–1.17)
April	202	33,435	0.93 (0.77–1.13)	47	0.88 (0.59–1.31)	155	0.95 (0.76–1.19)
May	243	33,806	1.11 (0.92–1.34)	70	1.29 (0.90–1.85)	173	1.06 (0.85–1.31)
June	257	33,068	1.20 (1.00–1.44)*	77	1.46 (1.03–2.08)*	180	1.12 (0.90–1.38)
July	268	34,574	1.20 (1.00–1.44)*	72	1.32 (0.92–1.89)	196	1.15 (0.93–1.42)
August	305	33,699	1.40 (1.18–1.67)**	106	1.96 (1.41–2.75)***	199	1.22 (0.99–1.50)
September	310	33,185	1.44 (1.21–1.72)***	94	1.79 (1.28–2.52)***	216	1.33 (1.08–1.63)*
October	324	31,516	1.59 (1.34–1.89)***	113	2.23 (1.61–3.12)***	211	1.38 (1.13–1.70)*
November	296	29,085	1.57 (1.32–1.88)***	95	2.04 (1.46–2.88)***	201	1.42 (1.16–1.75)***
December	342	29,826	1.77 (1.50–2.11)***	96	2.01 (1.44–2.83)***	246	1.70 (1.39–2.08)***
January–April	817	129,891	Reference	219	Reference	598	Reference
May–August	1073	135,147	1.26 (1.15–1.38)***	325	1.43 (1.20–1.70)***	748	1.20 (1.08–1.34)***
September–December	1272	123,612	1.64 (1.50–1.79)***	398	1.91 (1.62–2.25)***	874	1.54 (1.38–1.71)***

Note:IRRs calculated using generalized linear regression with Poisson error distribution.

Abbreviations: CI, confidence interval; IRR, incidence rate ratio.

*p < 0.05, **p < 0.001, ***p < 0.0001.

There were 749/3162 (24%) cases with comorbid ADHD. Younger cases born later in the year without ADHD displayed higher cumulative incidences of specific learning disorders than cases with ADHD (Figure 1B). The IRR for the youngest children, born in December, with comorbid ADHD was 1.59 (1.13–2.26, *p* = 0.008) and it was 1.84 (1.51–2.24, *p* < 0.0001) for those without comorbid ADHD (Table 2).

**TABLE 2:** Incidence rate ratios of specific learning disorder with and without comorbid ADHD by age 10 per birth month, pooled birth years 1996–2002

Birth month(s)	Comorbid ADHD		No comorbid ADHD	
	Cases (749)	IRR (95% CI)	Cases (2413)	IRR (95% CI)
January	54	Reference	156	Reference
February	46	0.92 (0.62–1.36)	147	1.02 (0.81–1.27)
March	59	1.05 (0.72–1.52)	153	0.94 (0.75–1.18)
April	55	0.99 (0.68–1.44)	147	0.92 (0.73–1.15)
May	60	1.07 (0.74–1.55)	183	1.13 (0.91–1.40)
June	58	1.05 (0.73–1.53)	199	1.25 (1.02–1.55)*
July	59	1.03 (0.71–1.49)	209	1.26 (1.02–1.55)*
August	66	1.18 (0.82–1.69)	239	1.48 (1.21–1.81)**
September	68	1.23 (0.86–1.77)	242	1.52 (1.24–1.86)***
October	74	1.41 (1.00–2.01)	250	1.65 (1.35–2.02)***
November	71	1.47 (1.03–2.10)*	225	1.61 (1.31–1.98)***
December	79	1.59 (1.13–2.26)*	263	1.84 (1.51–2.24)***
January–April	214	Reference	603	Reference
May–August	243	1.09 (0.91–1.31)	830	1.32 (1.19–1.47)***
September–December	292	1.43 (1.20–1.71)***	980	1.71 (1.54–1.89)***

*Notes*: For total number of children born, see Table 1. IRRs calculated using generalized linear regression with Poisson error distribution.

Abbreviations: ADHD, attention‐deficit/hyperactivity disorder; CI, confidence interval; IRR, incidence rate ratio.

*p < 0.05, **p < 0.001, ***p < 0.0001.

It was common to find overlapping other learning or coordination disorders in our sample, as 1360/3162 (43%) of children with specific learning disorders were also diagnosed with a speech, coordination, or mixed learning disorder. Younger children born in December, who were also diagnosed with speech, coordination, or mixed learning disorders, had an IRR of 1.52 (1.18–1.98, *p* = 0.002) for a specific learning disorder, whereas those with just a specific learning disorder displayed an IRR of 1.99 (1.59–2.52, *p* < 0.001; Table 3).

**TABLE 3:** Incidence rate ratios of specific learning disorder with and without comorbid speech, coordination or mixed disorder by age 10 per birth month, pooled birth years 1996–2002

Birth month(s)	Comorbid learning or coordination disorder		No comorbid learning or coordination disorder	
	Cases (1360)	IRR (95% CI)	Cases (1802)	IRR (95% CI)
January	98	Reference	112	Reference
February	88	0.97 (0.73–1.29)	105	1.01 (0.77–1.32)
March	94	0.92 (0.69–1.22)	118	1.01 (0.78–1.31)
April	88	0.87 (0.65–1.16)	114	0.99 (0.76–1.28)
May	113	1.11 (0.85–1.45)	130	1.12 (0.87–1.44)
June	104	1.04 (0.79–1.37)	153	1.34 (1.05–1.72)*
July	130	1.25 (0.96–1.62)	138	1.16 (0.90–1.49)
August	133	1.31 (1.01–1.70)*	172	1.48 (1.17–1.88)*
September	121	1.21 (0.93–1.58)	189	1.65 (1.31–2.09)***
October	137	1.44 (1.11–1.87)*	187	1.72 (1.36–2.18)***
November	117	1.33 (1.02–1.75)*	179	1.78 (1.41–2.27)***
December	137	1.52 (1.18–1.98)*	205	1.99 (1.59–2.52)***
January–April	368	Reference	449	Reference
May–August	480	1.25 (1.09–1.44)*	593	1.27 (1.12–1.44)**
September–December	512	1.46 (1.28–1.67)***	760	1.78 (1.58–2.00)***

*Notes*: For total number of children born, see Table 1. IRRs calculated using generalized linear regression with Poisson error distribution.

Abbreviations: CI, confidence interval; IRR, incidence rate ratio.

*p < 0.05, **p < 0.001, ***p < 0.0001.

## 4 DISCUSSION

We discovered a notable association between specific learning disorders and young relative age within the school year. Moreover, comorbid ADHD or other learning and coordination disorders did not influence the observed effect. To our knowledge, this was the first study to find an association between clinically diagnosed specific learning disorders and relative age in a population‐based sample. The findings were in line with studies that have explored school‐ or parent‐reported learning disorders and the need for special education in relatively young school children (Dhuey & Lipscomb, 2010; Gledhill et al., 2002; Martin et al., 2004). Our study has added further knowledge to the literature by examining specialized health care diagnoses and by clarifying the role of a suspected mediator, namely ADHD.

### 4.1 Strengths and weaknesses of the study

The strengths of this study were the nationwide sample of children diagnosed with specific learning disorders and the use of a uniform diagnostic system, based on ICD‐10 codes. The large sample size allowed us to carry out separate analyses of cases with, or without, comorbid ADHD and other learning or coordination disorders in mutually exclusive groups. Unlike some previous studies, we did not restrict our comparison to just the oldest and youngest groups in a school year but carried out analyses according to each birth month. Our study also showed that children born in the middle of the year, from May to August, had a higher risk of being diagnosed with specific learning disorders than their older peers.

This study had some limitations. First, the study only comprised diagnoses from specialized services, which meant that we did not have information on the relative age distribution among children with milder learning disorders who were not referred to those services. Second, our diagnostic data only went up to the age of 10. It could be considered that this made the sample less representative than a longer follow‐up period, but most children diagnosed with specific learning disorders in Finland are identified before the age of 10 (Arrhenius et al., 2018). Furthermore, the most recent data in this study is from the year 2012, however no major changes in the diagnostic procedures have been introduced after the year 2012 to this date. Third, we did not have information on the relative age distribution of pupils in special education and of pupils who had started school a year later, in the year they turned eight. However, no more than 1–2% of all pupils in Finland were held back at the time of the study, so this posed a low risk of substantial bias.

### 4.2 Possible explanations and comparison to other studies

Based on our data, we could speculate that children born later in the school year were slightly overdiagnosed and those born earlier were underdiagnosed. Yet, it needs to be pointed out that the total cumulative incidence in this study was 0.8% by the age of 10, which was low compared to the estimated 3%–8% lifetime prevalence of specific learning disorders in the population (Boyle et al., 2011; Landerl & Moll,  2010). This study included the more severe cases of specific learning disorders, because the children examined by school psychologists, who typically do not receive a specific diagnosis but might still receive special education, are not recorded in the Care Register for Health Care. The low cumulative incidence in the current study limited the conclusions of possible overdiagnosis of relatively young specific learning disorder cases to those diagnosed by specialized health care.

The potential explanations for our findings could lie in referral policies, with similar underlying mechanisms as in the relative age effect observed for ADHD. Younger children in class are, on average, less intellectually and emotionally mature than their classmates, and this likely causes them to behave and achieve below expectations for their school year level. As a result, this clustering of behavioral and academic difficulties produces proportionally higher rates of referrals to specialist services compared to older pupils in class. Relatively young children might also be more likely to undergo psychological testing in schools and, once referred, also in the specialist clinics. If they are tested more frequently, they will be referred by schools more frequently, and further receive more diagnoses in the specialist clinics. A Scottish birth cohort study (Lawlor et al., 2006) tested the cognitive skills of more than 12,000 children aged 7, 9, and 11 and found no significant differences in IQ due to their season of birth, especially after adjusting for the age when they started school. Those findings support the theory that the relative age effect for specific learning disorders is caused by the service system.

### 4.3 Implications and future research

Our findings have several implications. First, to prevent referral bias, teachers and other professionals screening for learning disorders should keep the educational disadvantages of the relatively young in mind when evaluating learning capabilities. Second, delaying the age at which children start school in Finland is becoming less common, which might disadvantage relatively young children. One study, based on survey and national register data in Denmark, demonstrated that immature girls, in particular, seemed to benefit substantially from being held back a year (Dee & Sievertsen, 2018). However, the benefits of increasing the proportion of children held back are not straightforward, because a greater age range within a classroom might paradoxically increase relative age effects, or shift the disadvantage of being youngest to other students (Whitely et al., 2020). Follow‐up studies on the well‐being of children held back are needed to explore the benefits and adversities of different policies in holding back immature pupils.

## 5 CONCLUSIONS

This study has demonstrated that even specific learning disorders, which are diagnosed with age‐standardized tools, are affected by the relative age effect. The findings underscore the importance of paying special attention to the vulnerable population of relatively young children in school grades. At the same time, medicalizing problems related to immaturity might lead to unwarranted diagnoses, which could result in labeling these children and generating feelings of reduced self‐worth. From a clinical point of view, the mounting evidence of relative age effects on psychiatric, cognitive, and other health‐related adversities indicates that children's relative age is an important factor to consider in pediatric care. From an educational perspective, increased flexibility in the time when less mature children start school might be justified, but further research on the topic is warranted.

## CONFLICT OF INTERESTS

The authors report no financial relationships or other conflict of interests relevant to this article to disclose.

## AUTHOR CONTRIBUTIONS

Dr. Arrhenius conducted the literature search and contributed to the study design, statistical analyses, interpretation of the results, and drafted the initial manuscript. Dr. Gyllenberg contributed to the study design, statistical analyses, interpretation of the results, and reviewed and revised the manuscript. Mr. Vuori contributed to the data analysis and interpretation; and reviewed and revised the manuscript. Dr. Tiiri contributed to the data analysis and interpretation; and reviewed and revised the manuscript. Ms. Lempinen contributed to the data analysis and interpretation; and reviewed and revised the manuscript. Prof. Sourander conceptualized the study, contributed to the study design, data collection and data interpretation, organized the funding of the study; and reviewed and revised the manuscript. All authors approved the final manuscript as submitted and agree to be accountable for all aspects of the work.

## ETHICS STATEMENT

Ethical approval for the study was provided by the Ethics Committee of the Hospital District of Southwest Finland and the National Institute for Health and Welfare (Registration number: THL/1803/5.05.00/2013). The participants were not contacted and therefore no informed consent was required according to Finnish data protection law.

## Data Availability

Individual participant data is not publicly available due to ethical restrictions. Summary level data can be provided by the corresponding author upon reasonable request.
